# State Anxiety Down-Regulates Empathic Responses: Electrophysiological Evidence

**DOI:** 10.3389/fnhum.2018.00502

**Published:** 2018-12-11

**Authors:** Pinchao Luo, Mengdi Zhuang, Jing Jie, Xiayun Wu, Xifu Zheng

**Affiliations:** ^1^School of Psychology, Center for Studies of Psychological Application, and Guangdong Key Laboratory of Mental Health and Cognitive Science, South China Normal University, Guangzhou, China; ^2^Center for Mental Health Education, Hainan University, Haikou, China

**Keywords:** state anxiety, empathy, ERP, N2, LPP

## Abstract

State anxiety is common in our life and has a significant impact on our emotion, cognition and behavior. Previous studies demonstrate that people in a negative mood are associated with low sympathy and high personal distress. However, it is unknown how state anxiety regulates empathic responses so far. Here, we recorded event-related brain potentials (ERP) from the experimental group who were in state anxiety and the control group when they were watching painful and neutral pictures. Participants in the experimental group and the control group were asked to do the same mental arithmetic problems. The only difference was that the experimental group had time restriction and was evaluated by the observer. The results showed that no significant N2 differentiation between painful and neutral stimuli was found in both groups. In contrast, LPP amplitudes induced by painful stimuli were significantly larger than that of neutral stimuli in the control group, but not in the experimental group. Our results indicate that state anxiety inhibit empathic responses from the early emotional sharing stage to the late cognitive evaluation stage. It provides neuroscientific evidence that one’s own emotional state will have an important impact on empathy.

## Introduction

Empathy is the capacity to share feelings and understand emotions or ideas of other people (Loggia et al., 2008; Singer and Lamm, 2009; Betti and Aglioti, 2016). It helps us understand others’ thoughts and emotional states, facilitate social communication and motivate prosocial behavior in our daily life (Acevedo et al., 2012). Due to the important role in individuals’ social interaction, empathy has become a research hotspot in the field of psychology and neuroscience (Chiao and Mathur, 2010; Fox et al., 2013; Keysers and Gazzola, 2014; Luo et al., 2015; Abraham et al., 2017). One interesting aspect of empathy is that how our own experiences and individual differences influence our ability to empathize with others. Some studies demonstrate that empathic responses are modulated by the relationship between the object and the observer, such as competitive relationship (Yamada et al., 2011; Wang et al., 2014; Gonzalez-Liencres et al., 2016), perceiving fairness of others (Singer et al., 2006), as well as in-group and out-group membership (Chiao and Mathur, 2010; Hein et al., 2010; Cheon et al., 2013). Other studies further find that the observer’s emotional states have significant impacts on empathic responses. People in a positive mood are more likely to focus on others’ needs and have a tendency to share others’ feelings (Eisenberg, 2000; Light et al., 2009). Conversely, people in a negative mood are associated with low sympathy and high personal distress (Liew et al., 2011).

State anxiety is an unpleasant emotional arousal when facing the dangers or threatening situations (Spielberger, 1972; Muris et al., 2008). It is a typical negative emotion induced by a cognitive appraisal of the threat (Lazarus, 2014) and has an impact on human’s emotion, cognition and behavior (Shackman et al., 2011). For example, a neuroimaging study conducted by Goldin et al. (2009) found that compared with anxiety participants, healthy controls had greater BOLD signal in regions implicated in attention processing (medial precuneus, left inferior parietal lobule, and right supramarginal gyrus), cognitive control (dorsolateral PFC, dorsal ACC), attention areas (bilateral dorsal parietal), visual feature detection (bilateral fusiform, superior temporal gyrus). Another behavioral study compared the capacity of emotion recognition of individuals who experienced aversive social events in an experimental group designed to increase state anxiety and to a control group (Auyeung and Alden, 2016). Participants were asked to rate the feeling of the targets in videos when discussing high school events in which they were either socially included or excluded. The results showed that participants who had been induced state anxiety were associated with greater accuracy recognition task.

Current models of empathy for pain involves affective and cognitive components (Decety and Jackson, 2004; Decety and Moriguchi, 2007). Affective empathy refers to an alternative or similar emotion for others, implicating emotional contagion, and affective sharing (Blair, 2005; Decety et al., 2010; Groen et al., 2013). Cognitive component is an advanced cognitive process that allows people to adopt others’ views to understand their emotional state, distinguish their feelings from one’s own, and integrate all information to guide interpersonal behavior eligibly (Michaels et al., 2014). Accordingly, recent ERP studies proved that the temporal dynamics of pain empathy consists of an early emotional sharing component (frontal N2) and a late cognitive evaluation component (centroparietal P3 or LPP) (Fan and Han, 2008; Escobar et al., 2014; Fan et al., 2014). State anxiety is common in our life, which affects our emotion and cognition. But so far, it is not known how state anxiety regulates empathic responses and at what stage of information processing this regulation occurs.

To explore this question, we recorded event-related brain potentials from the experimental group who were in state anxiety and the control group when they were watching painful and neutral pictures. Previous studies found that state anxiety could be induced by taking an intelligence test, especially being watched while performing the tasks (Chajut and Algom, 2003; Auyeung and Alden, 2016; Tomova et al., 2017). Here, we asked participants in the experimental group and the control group to do the same mental arithmetic problems. The only difference was that the experimental group had time restriction and was evaluated by the observer. State anxiety affects our emotional responses and cognitive processing (Hermans et al., 2014). Moreover, individuals who are in bad moods have difficulty in focusing on others’ situations and needs (Baroncohen et al., 2004). Thus, we hypothesized that empathic responses might not be observed at early emotional sharing (N2) and late controlled cognitive (LPP) stages in the experimental group, but not the control group. If this is the case, we anticipated state anxiety participants showed no difference in ERP responses between painful and neutral stimuli.

## Materials and Methods

### Participants

Thirty-eight healthy college students (19 males and 19 females, *M* = 20.87, *SD* = 2.17) were recruited in the study and signed the written informed consents. All participants were right-handed, with normal or corrected to normal vision, and reported no history of neurological or psychiatric disorders. They were randomly assigned to the experimental group or the control group. The experimental group included 19 participants (10 males and 9 females, *M* = 21.27, *SD* = 2.52), and the control group consisted of 19 participants (9 males and 10 females, *M* = 20.37, *SD* = 1.67). The study was approved by the Ethics Committee of South China Normal University. The procedure of the experiment was consistent with the principles of international researches involving human subjects in the Declaration of Helsinki (World Medical Organization, 1999).

### Visual Stimuli

Similar to those in previous ERP studies (Fan and Han, 2008; Decety et al., 2010), the stimuli consisted of 60 different digital color pictures showing one hand or two hands in painful and no-painful situations, 30 in each category. Painful pictures included situations such as a hand trapped in a door or cut by the scissors. Each painful picture was matched with a neutral picture that showed one or two hands in situations that, although similar in contexts, did not imply any pain. The stimuli were present at the center of a 17-in. color monitor with a white background. Each stimulus was a 10 cm × 7.6 cm (width × height) picture, subtending a visual angle of 7.5° × 5.5° at a viewing distance of 100 cm.

### Experimental Procedure

At the beginning of the experiment, participants were asked to fill out the State Anxiety Inventory (S-AI) in the State-Trait Anxiety Inventory (Spielberger, 1970) and Interpersonal Reactivity Index (IRI) (Davis, 1983). S-AI is a 4-point scale which consists of 20 items (10 positive items and 10 negative items) and are mainly used for assessing the individual’s fear, tension, anxiety, neurotic experience at present or recent a specific time. IRI is a 5-point scale which contains four subscales related to perspective taking (PT), fantasy scale (FS), empathic concern (EC) and personal distress (PD).

Before the ERP recording session, participants in the experimental group and the control group were asked to do the same mental arithmetic problems (add, subtract, multiply and divide of numbers short than 10). The only difference was that the experimental group had time restriction and was evaluated by the observer. To ensure that the manipulation induced state anxiety successful and continued through the experiment, each participant was required to rate anxiety level by a 7-point scale (1 = no anxiety, 7 = extremely high anxiety) after the mathematical task, as well as before and after the experiment.

The ERP recordings consisted of four blocks. Each block included 45 trials. Each trial started with a red fixation cross presented on a white background for 500 ms. Then, a blank white background appeared 1500–2000 ms, which was followed by a picture of the painful or neutral situation for 1000 ms. The order of the trial condition (pain, non-pain) was randomized. Finally, a question marked appeared on the screen and participants were asked to identify the content of the stimuli (painful or no-painful). The question marked was terminated either by pressing a button or after 3000 ms. After ERP recording, participants were asked to rate distress experienced by people in the pictures (other) and by themselves (self) using a 7-point scale (1 = no pain, 7 = great pain).

### ERP Recording and Analysis

EEG was continuously recorded using Brain Vision Recorder (BrainProducts) from 64 scalp electrodes that were mounted on an elastic cap in accordance to 10–20 system, with the references on the left and right mastoids and a ground electrode on the medial frontal aspect. Eye blinks and vertical eye movements were monitored with electrodes located above and below the left eye. The horizontal EOG was recorded from electrodes positioned 1.5 cm lateral to the left and the right external canthi. The EEG activity was amplified with a 0.01–00 Hz online band-pass filter and digitized at a 500 Hz sampling rate. All electrode impedances were kept below 5 Ω. The ERPs were computed off-line using Brain Vision Analyzer 2.0 software (Brain Products, Germany) (Fritsch and Kuchinke, 2013). The data under each condition were averaged separately off-line, and each epoch continued for 1200 ms with 200 ms before the picture onset for baseline correction. A band-pass filter of 0.01–0 Hz was applied offline to the EEG data. We used independent component analysis (ICA) to remove artifacts. Trials contaminated by eye blinks, eye movements, or muscle potentials exceeded ±100 V at any electrode were excluded from averaging.

The ERP components were chosen according to visual inspection of the grand-average data as well as prior the results of previous studies (Luo et al., 2013; Cheng et al., 2014; Choi and Watanuki, 2014). N2 (200–250 ms) component was observed predominately in the frontal and central regions, and LPP (350 –750 ms) component was distributed broadly in the scalp. Thus, we averaged the electrodes for each region of interest to obtain the mean amplitudes for N2 and LPP. The frontal (Fz, F1, F2, F3, F4) and central (Cz, C1, C2, C3, C4) regions were included for N2 analysis. The frontal (Fz, F1, F2, F3, F4), central (Cz, C1, C2, C3, C4) and parietal (Pz, P1, P2, P3, P4) regions were used for LPP analysis. Three-way ANOVA was conducted for N2 and LPP components. There was one between-group factor (group: experimental group and control group), and two within-group factors (stimuli: painful and neutral pictures; electrode distribution: frontal, central and parietal regions). The dependent variable was the mean ERP amplitude of each component at each electrode. Degrees of freedom were corrected with the Greenhouse-Geisser method.

## Results

### Behavioral Results

**FIGURE 1:**
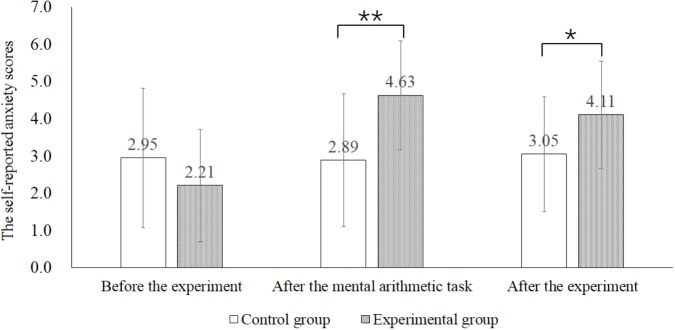
Self-reported anxiety scores in the control and experimental group before the experiment, after the mental arithmetic task and after the experiment. ^∗^*p* < 0.05, ^∗∗^*p* < 0.01.

The mean scores and standard errors for each subscale of IRI, S-AI and self-reported anxiety level were presented in Table 1. The results revealed that there were no significant differences between the experimental group and the control group on self-reported IRI scores and S-AI (*p*s > 0.05). We used independent sample *t*-test to compare the self-rating anxiety scores of the experimental group and the control group (Figure 1). There was no significant difference between the two groups before experiment (*t* = -1.336, *p* > 0.05). As expected, self-reported anxiety scores of the experimental group were significantly higher than that of the control group after the mental arithmetic task (*t* = 3.275, *p <* 0.05). Such significant difference lasted until the end of the experiment (*t* = 2.166, *p <* 0.05). The results indicated that the manipulation of state anxiety was successful.

**Table 1:** Descriptive statistic for the subscales (IRI and S-AI) and self-report anxiety scores in the experimental group and the control group.

	Experimental group		Control group	
	*Mean*	*SD*	*Mean*	*SD*
**IRI subscale**				
IRI-PT	13.05	2.72	12.10	3.05
IRI-FS	16.26	4.59	16.26	4.64
IRI-EC	18.11	2.96	17.21	3.46
IRI-PD	7.16	4.61	7.58	3.75
S-AI	39.37	7.88	37.16	8.45
**Self-report anxiety scores**				
Before the experiment	2.21	1.51	2.95	1.87
After the arithmetic task	4.63	1.46	2.89	1.79
After the experiment	4.11	1.45	3.05	1.54


*PT, perspective taking; FS, fantasy scale; EC, empathic concern; PD, personal distress; S-AI, state anxiety inventory.*

Table 2 showed the mean scores and standard deviation of subjective ratings of other-distress and self-distress. A 2 (group: experimental group and control group) × 2 (stimuli: pain and neutral) × 2 (perspective: other and self) ANOVA was conducted. There was a significant main effect of stimuli [*F*(1,36) = 560.160, *p* < 0.001, η^2^ = 0.841], group [*F*(1,36) = 7.073, *p* < 0.05, η^2^ = 0.010] and perspective [*F*(1,36) = 8.356, *p* < 0.05, η^2^ = 0.012]. A two-way interaction between stimuli and perspective [*F*(1,36) = 5.821, *p* < 0.05, η^2^ = 0.008] was significant. The simple effect of perspective was significant when viewing the painful pictures (*t* = 2.712, *p <* 0.05), but not viewing the neutral pictures (*t* = 2.011, *p >* 0.05). *Post hoc* analysis was conducted and confirmed that the rating scores of other-distress were significantly higher than that of self-distress in painful condition. Furthermore, the interaction between stimuli and group [*F*(1,36) = 4.079, *p* = 0.051, η^2^ = 0.006] was marginally significant. The simple effect of group was significant when painful pictures were presented (*t* = -2.389, *p <* 0.05). *Post hoc* analysis showed that the control group reported higher pain scores than the experimental group. However, such difference did not appear in neutral condition (*t* = -0.859, *p >* 0.05). The results suggested that anxious individuals paid less attention to painful stimuli and underestimated the distress, regardless of perspective.

**Table 2:** Descriptive statistic of subjective ratings of other-distress and self-distress when watching painful and neutral stimuli.

	Other-distress				Self-distress			
	Painful		Neutral		Painful		Neutral	
Group	*M*	*SD*	*M*	*SD*	*M*	*SD*	*M*	*SD*
Experimental group	5.04	1.00	1.20	0.02	4.52	1.46	1.14	0.19
Control group	5.72	0.79	1.28	0.47	5.38	1.08	1.24	0.42


### ERP Results

**FIGURE 2:**
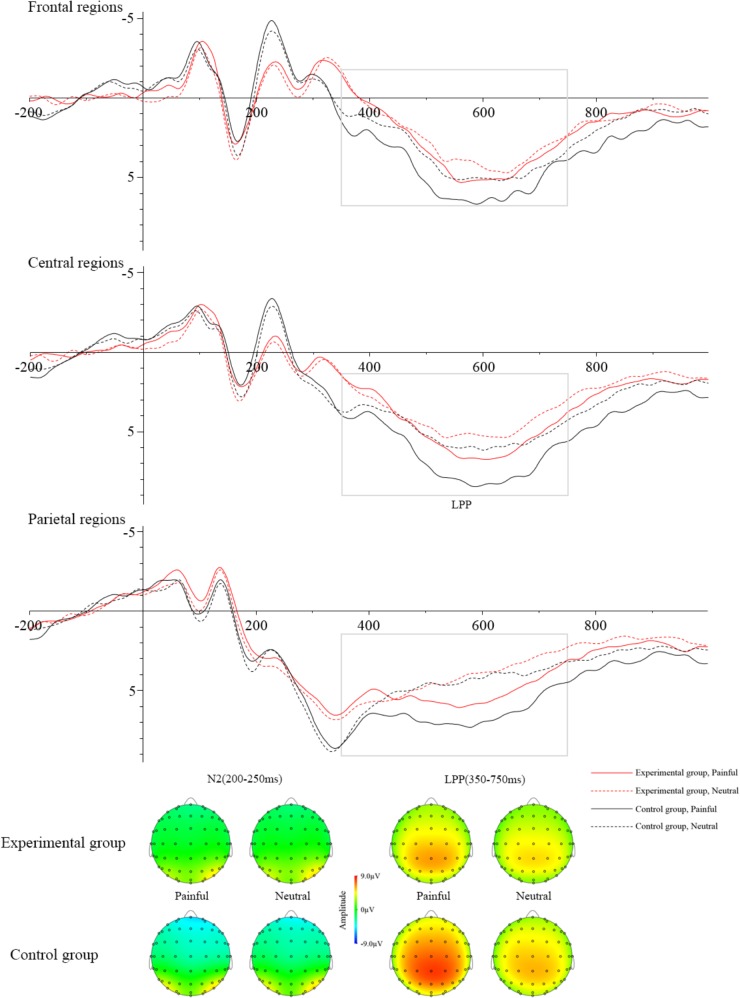
Grand averages of ERPs at Frontal regions, Central regions, and Parietal regions when watching painful pictures in the control group (black solid line), painful pictures in the experimental group (red solid line), neutral pictures in the control group (black dotted line), and neutral pictures in the experimental group (red dotted line). The voltage topographies illustrate the scalp distribution of N2 and LPP components.

Table 3 showed the mean amplitudes and standard error in each condition at N2 (200–250 ms) and LPP (350–750 ms). The averaged ERPs at frontal, central and parietal regions and the voltage topographies were presented in Figure 2. For the N2 component, N2 amplitudes were analyzed by a 2 (group: experimental group and control group) × 2 (stimuli: painful and neutral pictures) × 2 (electrode distribution: frontal regions and central regions) ANOVA. The analysis found a reliable main effect of group [*F*(1,34) = 6.064, *p* < 0.05), η^2^ = 0.114] and electrode distribution [*F*(1,34) = 32.887, *p* < 0.05, η^2^ = 0.068]. But the main effect of stimuli was not significant [*F*(1,34) = 0.258, *p* > 0.05]. *Post hoc* analysis found that when participants viewed stimuli including painful and neutral pictures, the experimental group elicited smaller negative deflection than the control group. Moreover, N2 amplitudes was induced larger negative deflection in the frontal region than that in the central region. No other interaction was found to be significant.

**Table 3:** Mean amplitudes (μV) and standard error in each condition at N2 (200–250 ms) and LPP (350–750 ms).

Group	N2 (200–250 ms)		LPP (350–750 ms)	
	Painful	Neutral	Painful	Neutral
**Experimental group**				
Frontal regions	–0.71 ± 3.89	–0.43 ± 4.00	3.83 ± 4.11	3.59 ± 4.43
Central regions	0.93 ± 3.15	1.14 ± 2.94	6.30 ± 4.35	5.65 ± 4.44
Parietal regions			6.20 ± 4.41	4.97 ± 4.21
**Control group**				
Frontal regions	–3.22 ± 3.45	–3.11 ± 2.99	6.52 ± 5.56	4.42 ± 4.66
Central regions	–1.09 ± 2.62	–1.10 ± 2.67	8.82 ± 3.79	6.34 ± 3.46
Parietal regions			8.08 ± 2.51	5.38 ± 3.28


LPP amplitudes were analyzed by a 2 (group: experimental group and control group) × 2 (stimuli: painful and neutral pictures) × 3 (electrode distribution: frontal regions, central regions, and parietal regions) ANOVA. The main effect of stimuli [*F*(1,36) = 47.781, *p* < 0.05, η^2^ = 0.033] and electrode distribution [*F*(2,72) = 7.130, *p* < 0.05, η^2^ = 0.045] were significant. The interaction between stimuli and group [*F*(1,36) = 14.384, *p* < 0.05, η^2^ = 0.009] was significant. The simple effect of stimuli was significant in the control group (*t* = 8.034, *p* < 0.05), but not in the experimental group (*t* = 2.092, *p* > 0.05). In the control group, pairwise comparison found painful stimuli elicited larger LPP than neutral stimuli. However, in the experimental group, pairwise comparison showed that there was no significant LPP difference between the painful and neutral stimuli. The interaction between stimuli and electrode distribution [*F*(2,72) = 12.238, *p* < 0.05, η^2^ = 0.001] was significant. The simple effect of stimuli was significant at frontal regions (*t* = 3.907, *p* < 0.05), central regions (*t* = 5.588, *p* < 0.05) and parietal regions (*t* = 7.583, *p* < 0.05). Post hoc analysis found that painful stimuli elicited larger LPP than neutral stimuli in all three regions (*p*s < 0.05). No other interaction was found to be significant. To investigate whether the electrophysiological activity elicited by the painful stimuli at late cognitive stage was correlated with subjective evaluation of distress, we calculated the correlation between the mean amplitudes of LPP component and subjective ratings of other-distress and self-distress. The results showed that the correlations were significant in the control group (other-distress: *r* = 0.487, *p* < 0.05; self-distress: *r* = 0.651, *p* < 0.05), but not in the experimental group (other-distress: *r* = -0.017, *p* > 0.05; self-distress: *r* = 0.413, *p* > 0.05).

## Discussion

This study examined the effect of state anxiety on empathic responses. The results showed that there was no significant N2 differentiation between painful and neutral stimuli in both groups. As for late centroparietal LPP component, the amplitudes were significantly larger while watching the painful pictures than neutral pictures in the control group. In contrast, no such late ERP responses were detected in the experimental group. This suggests the absence of pain empathic responses at late controlled cognitive stage when someone is in state anxiety. The behavioral results showed that the experimental group rated lower subjective scores for other-distress and self-distress than the control group when watching painful stimuli. The correlation analysis indicated that LPP was correlated with subjective evaluation of distress in the control group, but not in the experimental group. Our study provides neuroscientific evidence that state anxiety down-regulate empathic responses to others’ pain. This suggests that the regulatory factors of empathy include not only external relationship between the observer and the target, but also one’s own internal emotional state.

N2 results found that there was no significant difference in sensory information processing when watching painful and neutral stimuli, regardless of group type. This indicates that there is no difference in processing of painful and neutral stimuli in both the experimental group and the control group. Our result is inconsistent with previous studies which demonstrated that negative stimuli elicited significant smaller N2 amplitudes than neutral stimuli (Luo et al., 2013, 2014). N2 reflects affective sharing or affective arousal in current models of empathy for pain, using significant difference in ERP response between painful stimuli and non-painful stimuli as an index (Decety et al., 2010; Cheng et al., 2014). According to the subjective anxiety scores, the control group also had low anxiety although they were asked to complete the mathematical task without observation and time restriction. Negative emotional state is associated with low sympathy (Liew et al., 2011). One possible explanation is that affective sharing in both groups was hindered by state anxiety induced by the experimental environment (Klados et al., 2017). We also found that N2 amplitudes were less enhanced in the experimental group than in the control group. Previous research demonstrated that individuals with high state anxiety enhanced vigilance for threat information compared with individuals in low state anxiety (Bradley et al., 2000). Thus, the stimuli were processed more easily in the experimental group relative to the control group.

LPP can be used as objective indicator of attention activity when the stimuli are presented (Foti and Hajcak, 2008; Hajcak and Olvet, 2008) and is more positive in response to emotional stimuli than to neutral stimuli (Cuthbert et al., 2000; Weinberg et al., 2012). The increase of LPP is positively related to increased attention to stimuli (Weinberg and Hajcak, 2011). Our results showed that painful stimuli induced larger LPP amplitudes than neutral stimuli in control group, but not in the experimental group. Attention allocation may have contributed to the difference in the neural dynamics between the experimental group and the control group noted here. The Attentional Control Theory (ACT) suggests that anxiety impairs the efficient functioning of the goal-directed attentional system and reduces the ability of attentional control, especially in the presence of threat-related distracting stimuli (Eysenck et al., 2007; Rossi and Pourtois, 2017). According to ACT, anxiety has negative impact on the ability to allocate attentional and cognitive resources to painful stimuli and elicited small LPP amplitudes. That may be the reason why empathic responses disappear at LPP stage in the experimental group, but not in the control group. Besides that, we found the correlations between LPP amplitudes and subjective ratings of distress were significant only in the control group. High empathy is related to high ratings of distress while someone observes another individual receiving painful treatment (Loggia et al., 2008). In addition, the correlation between ERP responses and subjective feelings of others’ pain has been reported to be significant when empathic responses are obvious (Fan and Han, 2008; Cheng et al., 2012). Thus, our results indicate that empathic responses are down-regulated by state anxiety in the experimental group. However, empathic responses are still induced by pain stimuli in the control group at the late cognitive stage.

In conclusion, our study investigate how empathic responses are affected by one’s state anxiety. The results showed that no significant N2 and LPP difference was found when processing of painful and neutral stimuli in the experimental group, which suggested that state anxiety down-regulated the sensory processing elicited by the perception of other’s pain as well as cognitive regulatory processing elicited by the evaluation of other’s pain. This indicates that our ability to empathize with others in pain is hindered by state anxiety. Our results are consistent with previous researches which demonstrate that one’s own emotional state has an important impact on empathy (Light et al., 2009; Liew et al., 2011; Hermans et al., 2014). The current ERP study provides new neuroscientific viewpoints into how state anxiety affects the ability to experience empathy for pain. Unfortunately, there are some limitations in the present study. One limitation is that we manipulated the level of state anxiety through mental arithmetic task in different groups, unfortunately, we found that participants in the control group were induced low state anxiety. This may affect the results of our study. The most obvious is that empathic responses in N2 stage is not significant in controls. Another limitation is that we have not included a third group of participants with significant difference in the S-AI scores, which limits the generalization and application of the results. For future studies, it is recommended to take into account how to control the levels of state anxiety and establish experimental situation of more ecological validity, so that we can get more objective and scientific research results.

## Ethics Statement

This study was carried out in accordance with the recommendations of the Academic Committee of South China Normal University with written informed consent from all subjects. All subjects gave written informed consent in accordance with the Declaration of Helsinki. The protocol was approved by the Academic Committee of South China Normal University.

## Author Contributions

PL, MZ, and XZ designed the experiments and worked on the final version of the manuscript. JJ and XW collected and analyzed the data. All authors have read and approved the manuscript.

## Conflict of Interest Statement

The authors declare that the research was conducted in the absence of any commercial or financial relationships that could be construed as a potential conflict of interest.
